# Increased Basal and Alum-Induced Interleukin-6 Levels in Geriatric Patients Are Associated with Cardiovascular Morbidity

**DOI:** 10.1371/journal.pone.0081911

**Published:** 2013-11-14

**Authors:** Nathalie Compté, Karim Zouaoui Boudjeltia, Michel Vanhaeverbeek, Sandra De Breucker, Thierry Pepersack, Joel Tassignon, Anne Trelcat, Stanislas Goriely

**Affiliations:** 1 Institute for Medical Immunology (IMI), Université Libre de Bruxelles, Charleroi, Belgium; 2 Experimental Medicine Laboratory (Unit 222), Université Libre de Bruxelles, Hôpital A. Vésale, Montigny-Le-Tilleul, Belgium; 3 Service de Gériatrie, Hôpital Erasme, Bruxelles, Belgium; 4 ImmuneHealth, Charleroi, Belgium; South Texas Veterans Health Care System and University Health Science Center San Antonio, United States of America

## Abstract

**Background/Aim of the study:**

Low-grade systemic inflammation was suggested to participate to the decline of physiological functions and increased vulnerability encountered in older patients. Geriatric syndromes encompass various features such as functional dependence, polymorbidity, depression and malnutrition. There is a strong prevalence of cardiovascular diseases and related risk factors and chronic cytomegalovirus infections in the geriatric population. As these underlying conditions were proposed to influence the inflammatory state, the aim of this study was to assess their potential contribution to the association of geriatric syndromes with inflammatory parameters.

**Methodology:**

We recruited 100 subjects in the general population or hospitalized for chronic medical conditions (age, 23-96 years). We collected information on clinical status (medical history, ongoing comorbidities, treatments and geriatric scales), biological parameters (hematological tests, cytomegalovirus serology) and cytokines production (basal and alum-induced interleukin (IL)-1β and IL-6 levels). Using stepwise backward multivariate analyses, we defined which set of clinical and biological variables could be predictive for increased inflammatory markers.

**Principal Findings:**

We confirmed the age-associated increase of circulating IL-6 levels. In contrast to geriatric scales, we found history of cardiovascular diseases to be strongly associated for this parameter as for high IL-6 production upon *ex* *vivo* stimulation with alum.

**Conclusions:**

Association between low-grade inflammation and geriatric conditions could be linked to underlying cardiovascular diseases.

## Introduction

Geriatric patients are often affected by low-grade, chronic systemic inflammatory state. This process referred to as “inflamm-aging” was suggested to participate to the decline of physiological functions and increased vulnerability encountered in older patients [1]. Indeed, inflammatory markers, such as serum interleukin (IL)-6, C-reactive protein (CRP) or TNF-alpha are powerful predictors of morbidity and mortality in very old humans [2–5]. Furthermore, several reports indicate an association between inflammatory markers and geriatric conditions such as frailty, functional decline or depression [6–8]. 

Because of the overlap between geriatric syndromes and comorbidities, it is extremely difficult from the current literature to identify which clinical parameters are associated with increased inflammatory markers. Cardiovascular (CV) diseases are among the most prevalent comorbidities in geriatric patients [9]. CV diseases and related risk factors were proposed to influence the inflammatory state [10]. Other factors such as chronic cytomegalovirus (CMV) infections could also contribute to the association between geriatric conditions and low-grade inflammation [11,12]. The aim of this study was to assess the contribution of these underlying conditions in the association between inflammatory markers and common geriatric conditions (such as comorbidities, functional dependence, cognitive disorders, depression and malnutrition). For this purpose, we recruited old subjects in the general population or hospitalized for chronic medical conditions. We performed a comprehensive geriatric assessment (CGA) to identify comorbidities and common geriatric conditions. In this cross-sectional study, we also included younger subjects (with or without CV diseases) to assess the contribution of ongoing chronic co-morbidities in low-grade inflammation independently of age or geriatric conditions. 

We focused our analysis on plasmatic IL-6, a classical systemic inflammatory marker. In order to more specifically address the functional status of blood innate immune cells, we also monitored cytokine production upon *ex vivo* stimulation with Alum. This vaccine adjuvant is a classical activator of the NLRP3-dependent inflammasome [13]. On its own, it does not directly support transcriptional activation of pro-inflammatory genes [13]. However, if cells are in a pre-activated state that leads to accumulation of pro-IL1β (e.g. in response to Toll-like receptor (TLR) ligands), alum will induce the secretion of mature IL-1β and downstream IL-6 production. Hence, cytokine production in these conditions will indirectly reflect the global activation status of blood innate immune cells. 

## Materials and Methods

### Subjects

Between 2010 and 2012, 108 subjects aged between 23 to 93 years (65 women and 43 men) were enrolled in this cross-sectional study. The exclusion criteria were: CRP≥1 mg/dl, hepatic disturbance, presence of active cancer , autoimmune disease or infection, immunosuppression state, use of glucocorticoids, immunosuppressors or non steroid anti-inflammatory drugs (NSAID); advanced dementia (MMSE below 23 points[14]) was also excluded. Healthy young and old volunteers were recruited at the geriatric day ward of Erasme hospital among hospital and laboratory employees, volunteers of a non-profit seniors association (“Association pour le Soutien de l’Etude du Vieillissement”) or through public solicitation. Hospitalized volunteers were recruited from cardiology, neurology, rehabilitation or endocrinology units. For patients > 75 years, we assessed the risk of frailty (ISAR score >1 point) to recruit patients with geriatric conditions in geriatric unit (tertiary care at Erasme hospital, Brussels). The “Identification of Senior At Risk” (ISAR) score is a rapid scale performed at the emergency department which evaluates frailty and the risk of functional decline during hospitalization with 6 questions about dependence, previous hospitalization, eye troubles, memory problems and number of medications [15].

### Ethics statement

All subject signed an informed consent and the study received approval from Erasme hospital Ethics Committee (808 route de Lennik, B-1070 Brussels, Belgium, agreation n°OM021).

### Determination of clinical characteristics

All subjects were screened for underlying illnesses by direct questioning, medical archives and blood sampling. Social evaluation included determination of age, gender, home (private versus institution), and marital status. Clinical data comprised: smoking and alcohol habits, pneumococcal and influenza vaccine status, allergy, Body mass index (BMI), medical history, current treatment and reasons for hospitalization. Cardiovascular (CV) diseases were defined as history of stroke, myocardial infarct, cardiac insufficiency, cerebral vascular disease or atheromatosis assessed by carotid or leg Doppler echography and ischemic symptoms. CV risk factors were defined by the presence in the anamnesis of hypertension, type 2 diabetes, hypercholesterolemia or statin intake, infarct history or smoking. Osteoporosis was defined by self report.

For subjects > 75 years, we performed a CGA to identify comorbidity and common geriatric conditions. The polypathology and the severity of the medical problems were scored using the “Cumulative Illness Rating Scale-Geriatric” (CIRS-G). It is an instrument to quantify disease burden. It differentiates older adults with the highest risk and severity of infection with a markedly impaired vaccine response [16–18]. It comprises a comprehensive review of medical problems of 14 organ systems. It is based on a 0 to 4 rating of each organ system [19–21]. The “Geriatric Depression Scale” was used to assess the probability of depressed mood (GDS-15) in 15 questions [22–22]. The assessment of “Activities of Daily Living” (ADL) was made by using Katz’s scale. It includes the following items: bathing, dressing, transfer, toilet, continence and eating. Each task is graded on a 3-level scale (1 to 3 for Katz’s scale), where lower levels represent the absence of dependence and upper level the maximal dependence for the task [23]. Cognitive functions were assessed using the “Mini Mental State Examination” (MMSE). Possible scores range from 0 to 30 points, with lower scores indicating impaired cognitive function [14]. Nutritional status was assessed using the “Mini Nutritional Assessment” (MNA) [24,25]. A score ≥ 24 identifies patients with a good nutritional status. Scores between 17 and 23.5 identify patients at risk of malnutrition. These latter patients have not yet started to lose weight and do not show low plasma albumin levels but have lower protein-caloric intakes than recommended. A score < 17 indicates protein-caloric malnutrition. Pain was assessed using a visual analogical scale from 0 to 10 points. Maximal grip strength and fatigue resistance were measured using a Martin vigorimeter (Elmed Inc., Addisson, USA) as described previously [26,27]. Briefly, the shoulder is adducted and neutrally rotated, elbow flexed at 90°, forearm in neutral position and wrist in slight extension (0 to 30°). The subject is then asked to squeeze the large bulb of the vigorimeter as hard as possible. The highest of three attempts is noted as the maximal grip strength (in KPa). Afterwards, the subject is instructed to squeeze again the bulb of the vigorimeter as hard as possible and to maintain this maximal pressure; the time (in seconds) during which grip strength dropped to 50% of its maximum is recorded as fatigue resistance. This fatigue resistance test is highly reproducible in elderly subjects with ICC-values ranging respectively from 0.91 to 0.94 and from 0.88 to 0.91 for intra- and inter-observer reliability. An estimate of the total effort produced during the fatigue resistance test, defined as Grip Work, can be calculated as Grip Work = (Grip Strength × 0.75) × Fatigue Resistance. This parameter represents the physiologic work delivered by the handgrip muscles during the fatigue resistance test. When graphically represented, grip work is the area under the curve with grip strength in the vertical and time in the horizontal axis. All handgrip performance tests are executed with the dominant hand. 

We performed routine biochemical assessment to identify potential exclusion factors. CMV-specific IgG levels were determined by ELISA (ETI-CYTOK-GPLUS; Diasorin, P002033)

### Blood sample collection, management and ex vivo stimulation

Venous blood samples (55ml) from all subjects were collected in pyrogen-free, heparinized tubes between 11.00 and 13.00. After 2 h (because of transport), blood samples were centrifuged; plasma were collected and stored at -80°. Basal IL-6 was measured by ELISA (quantikine immunoassay, R&D systems).

 Blood was diluted 2-fold with sterile RPMI-1640 and incubated in the presence or not of alum (Alum hydroxide and magnesium hydroxide, 77161, Pierce/Thermo Fischer, 500µg/ml) at 37°C and 5% CO2. After 18h, cell-free supernatants were collected and stored at -80° to measure cytokine production. IL-1β and IL-6 levels were determined using ELISA kits (duoset, R&D systems). A control sample was placed in all ELISA plates to assess the internal quality of our tests.

### Statistics

The SigmaStat® software package version 3.5 (Jandle Scientific) was used for multivariate analyses and GraphPad prism 5® software for univariate analyses and Mann-Whitney rank sum test. For statistical analyses, cytokine concentrations were log_10_-transformed in view of their non parametric distribution.

In order to identify clinical and biological factors that could be significantly associated with cytokine levels, we performed univariate analyses (depicted by Pearson’s coefficient). For all volunteers, these parameters comprised: age, gender, CV diseases and risk factors (smoking, arterial hypertension, type 2 diabetes, cholesterol levels, BMI), monocytes and white blood cell counts (WBC), depression, creatinin clearance and CMV status. For individuals > 75 years, geriatric evaluations (MNA, KATZ, GDS, MMSE, CIRS scales, grip strength and osteoporosis) were also included in this analysis.

In order to define independent predictive factors of inflammation, we tested several models by stepwise backward multi-linear regression analyses. To decrease the risk of over-fitting, no more than 8 or 5 variables were included at a time for the entire study group (n=100) or for older patients (n=52), respectively. 

Katz and MNA (cut off values: ≥ 8, ˂23.5, ˃1 respectively) and clinical variables were treated as dichotomous variables while other data were continuous. A probability level of p<0.05 was considered to be significant. 

## Results

### Characteristics of the enrolled individuals

In the entire study population, 8 subjects were excluded because CRP values exceeded 1mg/dl and 100 subjects were included in the analyses. The demographic, clinical and biochemical characteristics of the entire group are presented in table 1. Fifty two subjects were older than 75 years. We performed a comprehensive geriatric assessment in this subgroup. The characteristics of old individuals with ISAR>1 (n=25) or ISAR≤1 point (n=27) are presented in tables 2-4. As expected, in patients with ISAR>1 point, decreased grip strength, depression state (GDS), comorbidity burden (CIRS-G), dependence (Katz scale), cognitive troubles (MMSE) and impaired nutritional status (MNA) were more prevalent in comparison to individuals with ISAR≤1 point. However, 4/25 individuals in the group ISAR>1 point did not present any geriatric conditions (Katz >8 points, MNA ≤ 23.5 points or GDS > 5 points). In the group ISAR ≤1 point, one patient had an MNA score at 18.5 points and two patients had a GDS score at 6 points.

**Table 1:** Main characteristics of the study group.

	**Entire group**
N	100
Age (year)	66.4 (23 - 93)*
Gender M/F	43/65
BMI (kg/m^3^)	24.9 (17-46)*
Active smokers (%)	11
Hypertension (%)	50
Type 2 diabetes (%)	12
Hypercholesterolemia (%)	56
Cardiovascular diseases (%)	29
CMV seropositivity (%)	55

* Median (range)

**Table 2:** Demographic characteristics and comorbidities of old individuals with ISAR score ≤ 1 point and > 1 point.

	ISAR≤1	**ISAR>1**
N	27	25
Recruitment type	Ambulatory	Hospitalized
Age (years)	80 (76-88) *	83 (73-90) *
Gender (M/F)	9/18	8/17
BMI (kg/m^2^)	25.4 (19-32)*	24.7 (18-34)*
Active smokers (%)	0	8
Hypertension (%)	48	28
Type 2 diabetes (%)	0	28
Hypercholesterolemia (%)	74	60
Cardiovascular diseases (%)	3.7	60
Osteoporosis (%)	14.8	40

* Median (range)

**Table 3:** Geriatric characteristics of old individuals with ISAR score ≤ 1 point and > 1 point.

	ISAR≤1	**ISAR>1**
ISAR (score)	0 (0-1)	3 (2- 4)
GDS (score)	1 (0- 2)	5 (2-5)
Katz (score)	6 (6-7)	9 (7-12)
MMSE (score)	29 (28- 29)	26 (24- 28)
MNA (score)	26.75 (25.6-27.8)	20 (17.5- 22.5)
Vigorometer Force (kPa)	30 (17- 45.5)	10 (0-24)
Vigorometer ; fatigue resistance (sec)	20 (10- 40)	0 (0-10)
CIRS-G (category number)	6 (3.5 -6.5)	9 (7-10)
CIRS-G (global score)	9 (6.5-11)	19 (15-22)
CIRS-G (severity index)	1.6 (1.4-2)	2.2 (1.72 -2.33)

Median (range)

**Table 4:** Biochemical characteristics of old individuals with ISAR score ≤ 1 point and > 1 point.

	ISAR≤1	**ISAR>1**
Cholesterol (mg/dl)*	202 (182 -223)	187 (173 -206)
Ferritin (mg/dl)*	107 (60-184)	176 (112 -275)
Prealbumin (mg/dl)*	25 (22-28)	19 (17-24)
CMV seropositivity (%)	48	64

* Median (range)

### Occurrence of cardiovascular diseases is independently associated with high circulating IL-6 levels

We analyzed basal plasma IL-6 levels rather than spontaneous secretion by isolated PBMC or monocytes as it also reflects the contribution of other potential sources such as adipocytes, muscle or endothelial cells. 

Using univariate analyses, we first attempted to identify relevant associations between clinical and biological parameters on one hand and basal IL-6 levels on the other. We found a significant association for monocytes (R^2^=0.8; p= 0.01) and WBC counts (R^2^=0.12; p=0.001), age (R^2^= 0.05; p=0.03), CV risk factors (R^2^=0.08; p=0.01) and diseases (R^2^=0.14; p=0.0007), type 2 diabetes (R^2^=0.13; p=0.013) and a trend for hypertension (R^2^=0.08; p=0.06).

To assess the relative contribution of these variables, we next performed multivariate analysis (summarized in Table 5) including age, gender, cardiovascular risk factors and diseases, WBC and monocytes counts, creatinin clearance and CMV status. Age, CV diseases and WBC counts appeared to be associated with basal IL-6 levels. IL-6 values were then normalized to WBC counts and we performed the same multivariate analysis. Once again, age and CV diseases were found to be significantly associated with basal IL-6 levels/WBC ratio. 

**Table 5:** Multivariate analyses for basal IL-6 levels in the whole study group.

**N=100**	**R^2^ ; F value**	**Standardized Coefficient**	**p value**
**Log_10_ IL-6^1^**	R^2^=0.31; F=10.93		
WBC		0.363	<0.001
CV diseases		0.319	0.002
Age		0.255	0.0013
**Log_10_ IL-6/WBC ratio ^2^**	R^2^=0.31; F=10.93		
CV diseases		0.35	0.001
Age		0.232	0.03

1 adjusted for age, gender, CV risk factors and diseases, WBC and monocytes counts, creatinin clearance, CMV status.

2 adjusted for age, gender, CV risk factors and diseases, creatinin clearance, CMV status.

**Figure 1:**
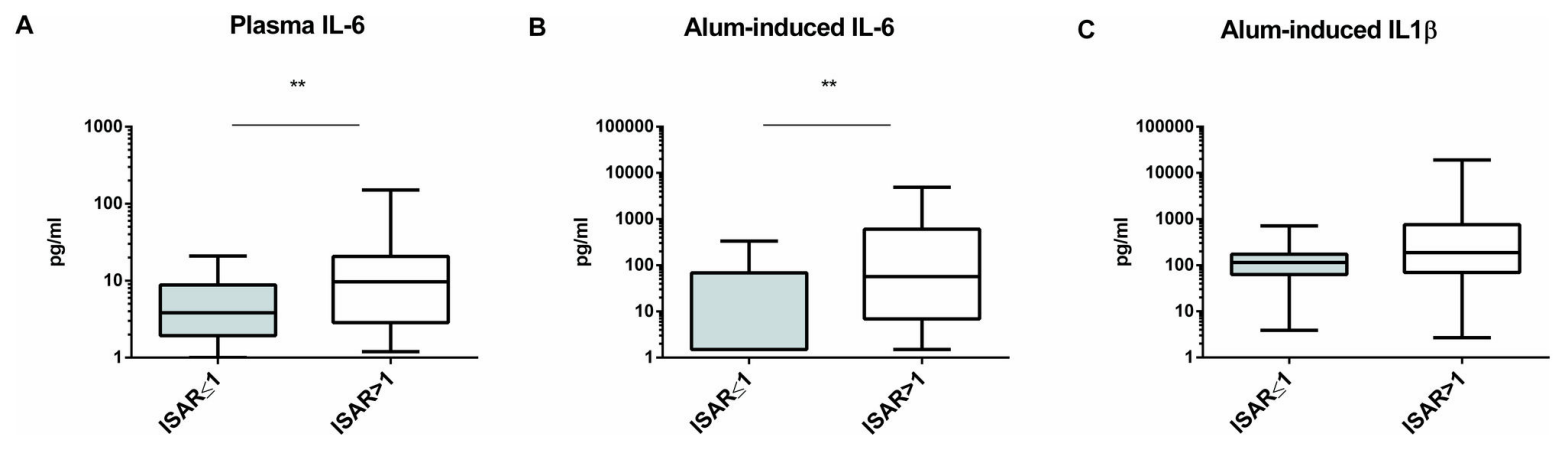
Inflammatory markers in individuals with ISAR ≤1 and **>1** **point**. (A) Plasmatic IL-6 levels were analyzed by ELISA. (B-C) Whole blood samples were incubated with alum (500µg/ml) for 18h. Cell-free supernatants were collected and analyzed for IL-6 and IL-1β levels by ELISA. ISAR>1 point, n=27; ISAR≤1 point, n=25. For statistical analysis, we used the Mann-Whitney rank sum test *p<0.05; **p<0.01.

Secondly, we assessed the association between geriatric conditions and low-grade inflammation. Geriatric scales are only validated in aged individuals. Hence, to assess the association between inflammatory state and geriatric conditions, we restricted our analyses to subjects >75 years of age. We compared plasma IL-6 levels between older individuals with ISAR>1 and ISAR≤1 point. IL-6 levels were significantly increased in this second group (Figure 1). To take into account possible confounding variables, we performed univariate analyses. No geriatric conditions (MMSE, CIRS-G and grip strength) were significantly correlated with this inflammatory parameter but there was a trend for Katz, MNA and GDS. As for the entire study group, we observed a significant association between CV diseases (R^2^=0.1; p=0.04), type 2 diabetes (R^2^=0.13; p=0.02) and plasma IL-6 as well as a trend for CV risk factors (R^2^=0.08; p=0.07). We performed multivariate analyses with age, CV risk factors, gender and geriatric scales. As geriatric scales influence each other, we analyzed separately MNA, GDS or MMSE scores in multivariate analysis. No geriatric score appeared as a significant predictive factor (cfr table 6). 

**Table 6:** Multivariate analyses for basal IL-6 levels in subjects **> 75 years**.

**N=52**	**R^2^ ; F value**	**Standardized Coefficient**	**p value**
**Log_10_ IL-6^1^**	R^2^=0.13; F=6.5		
Age		0.03	0.014
**Log_10_ IL-6^2^**	R^2^=0.14; F=7		
Age		0.03	0.011
**Log_10_ IL-6^3^**	R^2^=0.13; F=6.5		
Age		0.03	0.014
**Log_10_ IL-6/WBC ratio^4^**	R^2^=0.15; F=7.7		
**Age**		0.04	0.008
**Log_10_ IL-6/WBC ratio^5^**	R^2^=0.16; F=8.1		
**Age**		0.04	0.007
**Log_10_ IL-6/WBC ratio^6^**	R^2^=0.15; F=7.7		
**Age**		0.04	0.008

1 adjusted for Katz, age, gender, CV risk factors and WBC counts.

2 adjusted for MNA, age, gender, CV risk factors and WBC counts.

3 adjusted for GDS, age, gender, CV risk factors and WBC counts.

4 adjusted for Katz, age, gender, CV risk factors and diseases.

5 adjusted for MNA, age, gender, CV risk factors and diseases.

6 adjusted for GDS, age, gender, CV risk factors and diseases.

### CV diseases rather than geriatric conditions are associated with cytokine production upon alum stimulation

Incubation of whole blood with alum led to the induction of IL-1β and downstream IL-6 production in most donors. In univariate analyses, CV risk factors (R^2^=0.06, p=0.003) and diseases (R^2^=0.06; p=0.01), hypertension (R^2^=0.07; p=0.007), WBC counts (R^2^=0.07; p=0.01) were significantly associated with IL-1β production post-alum and there was a trend for type 2 diabetes (R^2^=0.03; p=0.07) and for monocytes counts (R^2^= 0.03; p=0.07). For IL-6 production upon alum stimulation, CV risk factors (R^2^=0.06; p=0.02), type 2 diabetes (R^2^=0.04; p=0.048), CV diseases (R^2^=0.05; p=0.02), monocytes counts (R^2^=0.06; p=0.02) were significant predictive factors. For this parameter, we also observed a trend for CMV status (R^2^=0.03; p=0.09). 

We then performed multivariate analyses to assess the relative contribution of these factors. CV diseases were significantly associated with both alum-induced IL-1β and IL-6 levels (table 7). The same analyses were performed after normalization of cytokine levels with WBC counts. Once again, CV diseases were found to be significantly associated with alum-induced IL-1β and IL-6 levels/WBC ratio. CMV status was also found to be associated with alum-induced IL-6/WBC ratio. We confirmed that alum-induced IL-1β/WBC ratio was associated with CV risk factors. We next compared the levels of inflammatory markers between older subjects with ISAR score ≤ 1 point and > 1 point. Alum-induced IL-6 levels but not IL-1β levels were significantly increased in this second group (Figure 1). In these older subjects, univariate analyses confirmed that CV risk factors (R^2^=0.19; p=0.001) and CV diseases (R^2^=0.17; p=0.002) were significant predictive factors for IL-1β production. Similarly, for IL-6 production upon alum stimulation, CV risk factors (R^2^=0.17; p=0.002), CV diseases (R^2^=0.19; p=0.001) and CMV status (R^2^=0.08; p=0.03) were significant predictive factors. We found a positive trend for MNA, MMSE and GDS for IL-6 upon alum stimulation. 

**Table 7:** Multivariate analyses of alum-induced cytokine levels in the whole study group.

**N=100**	**R^2^ ; F value**	**Standardized Coefficient**	**p value**
**Log_10_ Alum-IL-6^1^**	R^2^=0.08; F=9		
CV diseases		0.29	0.003
**Log_10_ Alum-IL-1^1^**	R^2^=0.13; F=7.1		
CV risk factors		0.26	0.01
WBC		0.21	0.03
**Log_10_ Alum-IL-6/WBC ratio^2^**	R^2^=0.12 ; F=6.3		
CV diseases		0.29	0.004
CMV		0.19	0.048
**Log_10_ Alum-IL-1/WBC ratio^2^**	R^2^=0.07 ; F=7.5		
CV risk factors		0.27	0.007

1 adjusted for age, gender, CV risk factors and diseases, WBC and monocytes counts, creatinin clearance, CMV status.

2 adjusted for age, gender, CV risk factors and diseases, creatinin clearance, CMV status.

We performed multivariate analyses with age, gender, CV risk factors and diseases, CMV status, WBC counts and geriatric scales (table 8). As geriatric scales influence each other, we performed the same multivariate analyses replacing MNA score by GDS or MMSE score for IL-6 upon alum stimulation. In contrast to geriatric scales, CV risk factors were found to be significant predictive factors for IL-6 and IL-1β levels. Taken together, we observed that association between low-grade inflammation and geriatric conditions could be linked to the presence of cardiovascular diseases.

**Table 8:** Multivariate analyses of alum-induced cytokine levels in subjects > 75 years.

**N=52**	**R^2^ ; F value**	**Standardized Coefficient**	**p value**
**Log_10_ Alum-IL-1/WBC ratio^1^**	R^2^=0.18 ; F=10.6		
CV risk factors		0.42	0.002
**Log_10_ Alum-IL-6/WBC ratio^1^**	R^2^=0.35 ; F=8.7		
CV risk factors		0.45	<0.001
CMV		0.34	0.005
Gender		0.26	0.03

1 adjusted for age, gender, CV risk factors, CMV and GDS or MNA or MMSE.

## Discussion

Previous studies on inflammation in older individuals and geriatric patients yielded conflicting conclusions. The main difficulty in the interpretation of the results comes from the heterogeneity of the population and differences in the classification, definitions and inclusion/exclusion criteria used. A summary of such studies is shown in Table S1. We included main study parameters (size of the group, age groups, inclusion criteria, clinical data, design and statistical methods used) and their conclusions. Increased inflammatory markers are generally associated with age, CV comorbidities and geriatric conditions. However, when confounding factors are taken into account, the relative contribution of these parameters is less clear. Herein, we took particular care to characterize the clinical parameters of the enrolled subjects. As CV disorders are associated with low-grade inflammation and as healthy old individuals might present underlying CV disorders without any symptoms, we included younger subjects (with or without CV diseases) to assess the contribution of ongoing chronic co-morbidities in low-grade inflammation independently of age or geriatric conditions. Indeed, in enrolled younger subjects (mean age: 49 years), proportion of CV diseases or risk factors (hypertension: 38%, type 2 diabetes: 10%, hypercholesterolemia: 63% and ongoing CV diseases: 27%) was comparable to that observed in older individuals. We observed that the strongest predictive factors for increased IL-6 plasmatic levels were the history of CV diseases or risk factors and age. A previous study indicated that IL-6 levels were associated with age but not with CV risk factors (assessed by BMI, blood pressure or lipidemia) in a 20-84 year old healthy population. However, the authors did not report the occurrence of CV diseases in their study population [7,28]. Forsey et al. also observed an increase of IL-6 with age and poor health status (using SENIEUR, OCTO and NONA criteria) but they did not characterize their group for ongoing comorbidities [29]. Hence, as suggested previously [10], our findings support the notion that part of age-associated inflammatory state could be linked to accumulation of CV risk factors and morbidities in the geriatric population. In another study, high inflammatory markers (in particular basal IL-6) were found to be predictive for the development of CV events within a 3-year period in older well-functioning individuals [30]. In people without known CV diseases, CRP level is also an important predictive factor for CV events [31]. Furthermore, two large mendelian randomized studies showed that polymorphisms of the IL-6R are associated with reduced CRP levels and the risk of coronary artery diseases. However, both studies showed increased concentrations of IL-6 levels that seem in contradiction with a causal role of IL-6. This paradox might be resolved by the fact that both IL-6R polymorphisms seem to be associated with downregulation of IL-6R signaling and that increased IL-6 levels could be a result of a feedback loop to increase IL-6 signaling [32,33]. A meta-analysis of prospective studies involving white populations confirmed that polymorphisms of the IL-6 gene were also associated with the development of coronary artery diseases [34]. 

We observed that IL-1β and IL-6 production by blood cells in response to alum was also strongly associated with CV diseases and risk factors. This result suggests that in these patients, circulating innate immune cells could be in a pre-activated state. Indeed, CV risk factors such as hypercholesterolemia, high saturated fat diet, obesity and hyperglycemia are known to promote inflammation through different pathways such as the endoplasmic reticulum (ER) stress, the inflammasomes and the TLRs [3,35,36]. ER stress and TLR pathways both lead to transcriptional activation of inflammatory genes such as pro-IL1β that could account for the association between high IL-1β and IL-6 production in response to alum and CV diseases that we observed. However, the design of our study does not allow us to establish a causal links between CV diseases and low-grade inflammation. Coronary artery diseases, high BMI and atherosclerosis are associated with an increase in circulating inflammatory monocytes (CD14^dim^CD16^+^). Furthermore, the proportion of intermediate monocytes (CD14^++^CD16^+^) is also associated with increased CV risk factors and is predictive of CV events and poor outcome [37,38]. In comparison to classical monocytes (CD14^++^CD16), this subset displays an activated phenotype, reduced telomere length and produces high amounts of proinflammatory cytokines [39,40]. It would therefore be important to determine the responsiveness of these cells to alum in the context of CV diseases. 

Along this line, chronic CMV infection was also associated with modulation of monocyte functions [41] that could account for the association between CMV status and IL-6 levels upon alum that we observed. Chronic infection with CMV is seen as a major factor that could influence exhaustion of memory T lymphocytes in older individuals. CMV seropositivity is part of the “immune risk phenotype” (IRP) and is associated with increased all-cause mortality [42]. It has been suggested that immune response to persistent CMV infection could also participate to the establishment or maintenance of inflamm-aging processes [2,42]. Several studies previously reported an association between IL-6 levels and CMV status. In patients with coronary artery diseases, Blanckenberg et al. observed that CMV titers were correlated to IL-6 levels and together could predict cardiac mortality [43]. In community-dwelling post-menopausal women, mean IL-6 levels were found to be higher in CMV+ subjects but significance was lost after adjustment for confounding factors [11,44]. Interestingly, in the “Hertfordshire Ageing study” group, increase in plasma IL-6 levels upon aging was similar in seronegative, seropositive volunteers and volunteers who became seropositive for CMV, arguing against a central role of CMV infection in this process [45]. We identified CMV status as an independent predictor of IL-6 production upon alum stimulation but not for other inflammatory parameters, suggesting that CMV could directly or indirectly influence the capacity of the immune cells to produce this cytokine. It would be important to define the potential mechanistic links responsible for these observations. 

In our study, geriatric patients with ISAR>1 point display increased basal and alum-induced IL-6 production compared to old individuals with ISAR<1 point. However, we did not find any significant association between geriatric conditions (assessed by Katz, MNA, GDS, CIRS-G, MMSE, Grip strength) and low-grade inflammation. Studies that have identified association between frailty and high IL-6 levels generally used criteria developed by Fried et al. (slow gait speed, low physical activity, unintentional weight loss, self-reported exhaustion and muscle weakness) [46]. Initial studies indicated an association between elevated CRP levels and frailty after exclusion of CV diseases and diabetes [47]. However, association between increased IL-6 and frailty was less clear in other studies. For example, in the “Longitudinal Aging Study Amsterdam” (LASA), IL-6 was not associated with frailty parameters [48]. While IL-6 could be predictive for frailty in a cohort of women aged 70 to 79 [11], increased IL-6 levels were not found to be predictive for the later development of frailty in the “Hertfordshire Ageing study” (HAS, 10 year follow-up, age between 65-70 years) [49]. The use of NSAID and corticoids, the heterogeneity of the population and follow-up bias because of mortality and various frailty criteria could partly explain these discrepancies.

Dependency is a direct expression of frailty. In institutionalized subjects older than 65 years old selected for cerebral vascular diseases, a decline in activities of daily living (Katz ADL and Barthel index) was associated with high IL-6 levels [8]. Gonzalo-Calvo et al. showed the same results in institutionalized volunteers [8,50]. However, variability in interpretation of Katz score hampers interpretation and comparison of these studies with the present one [51]. 

Muscle strength and walking speed are major phenotypic criteria of frailty. Several studies used this parameter to assess the link between frailty and inflammation. Indeed, Schaap et al. observed that higher IL-6 and CRP levels were associated with decreased muscle strength in a cohort of independent individuals over 55 years after adjustment for confounders such as age, depressive symptoms, chronic diseases and cognitive troubles [52]. Similar conclusions were also previously reached by Ferruci et al. in community-dwelling women (above 65) [53]. In a cohort of healthy subjects between 20 to 72 year old, Blain and colleagues observed a similar association between IL-6 levels and walking performance but not with muscle strength [54]. Interestingly, Bautmans et al. showed that while better grip strength and fatigue resistance were associated with higher IL-6 levels in well-functioning subjects, this was not the case for nursing home patients and hospitalized patients [26,55,56]. Taken together, these data indicate that association between inflammation and grip strength is highly dependent on the population under study and might reflect variations in important confounding factors. In particular, CV diseases and underlying pathological conditions (such as atherosclerosis) are likely to participate to the development of frailty and reduced multi-organ physiological reserve in old age. Hence, CV diseases, frailty and geriatric conditions are probably deeply intertwined. 

 Cognitive and psychological factors are other important aspects of the geriatric patient. As these factors also participate to the general health status of the patients and could impact their immunological profile, we used a CGA to characterize our patients [57,58]. Some studies found an association between depressive or stress symptoms and IL-6 levels [6,7,59]. Meta-analysis confirmed the association between IL-6, CRP and IL-1β levels and depression. However, it was not significant anymore once age or medication intake were taken into account [60]. In the context of this study, for ethical reasons and accuracy of the other geriatric scales, we did not recruit patients presenting with strong cognitive impairment. The potential association of these clinical parameters and inflammation is therefore probably underestimated. Several studies have shown an association with cognitive decline and IL-6 [61,62] but most studies observed an association with vascular dementia but not Alzheimer’s dementia [63,64], suggesting that underlying CV conditions could be responsible for such association. However, other studies that included patients with low MMSE scores did not detect an association for TNFα and IL-6 levels [65–67]. 

One limit of our study is the small size of the group given its heterogeneity. We performed numerous comparisons that increase α error of the statistical analyses. As we recruited patients that were hospitalized, acute medical conditions could bias inflammatory markers. To reduce this potential effect, data were collected just before discharge to home. Although conclusions were similar when the whole group was analyzed (not shown), we limited evaluation of geriatric scales to subjects above 75 years of age, as these clinical parameters might not reflect the same underlying conditions in younger patients. As we included younger and older individuals with chronic diseases, we cannot fully assume that the impact of these diseases on physiological and immune functions will be the same in both age groups. Transversal studies do not take into account the evolution of clinical and immunological parameters. We did not specifically look at periodontal diseases known to be associated with chronic inflammation [68]. 

Despite these limitations, we identified a clear association between CV diseases and inflammatory parameters suggesting that among clinical parameters, these factors have the largest impact on the development of low-grade inflammation observed in the geriatric population. 

## Supporting Information

Table S1 **Summary of studies that looked at inflammatory markers in old age.** A) Correlation of plasma IL-6 levels with age, comorbidities and frailty. B) Inflammatory markers as predictive factor for mortality in old age.
(DOC)
