# Supplementary material for: Increased Basal and Alum-Induced Interleukin-6 Levels in Geriatric Patients Are Associated with Cardiovascular Morbidity
Source: PLoS One. 2013 Nov 14;8(11):e81911. doi: 10.1371/journal.pone.0081911 (PMC3828251; doi:10.1371/journal.pone.0081911)
Supplement: Table S1 — Summary of studies that looked at inflammatory markers in old age. A) Correlation of plasma IL-6 levels with age, comorbidities and frailty. B) Inflammatory markers as predictive factor for mortality in old age. (DOC) [file pone.0081911.s001.doc]

**Table S1 : Summary of studies that looked at inflammatory markers in old age.**

1. **Correlation of plasma IL-6 levels with age, comorbidities and frailty**

| Reference | Age | Group size | Clinical characteristics | Design | Statistics | Outcomes |
| --- | --- | --- | --- | --- | --- | --- |
| [1] | y = 18-39 m=40-59  o=60-84 | y=96 m=31 o=35 | H | T | ANOVA, MVA adjusted for age and CV risk factors | Basal IL-6 increases with age and not with cardiovascular factors. |
| [2] | O=86-94  Y=32-59 | N=136 | OCTO, NONA,SENIEUR | T | Wilcoxon Mann-Whitney | Basal IL-6 increases with age and comorbidities |
| [3] | 20-92 | n=70 | C | T | Wilcoxon Mann-Whitney rank sum test and linear regression without adjustement | TNFα and TGFα levels are higher in old subjects in contrast to IL-10 and IL-6 levels |
| [4] | O=65-85  y= 20-30 | y=20 o=26 | S | T | Wilcoxon Mann-Whitney rank sum test | IL-6 does not increase with age |
| [5] | 40-80 | n=397 | C | T | ANOVA | Basal IP-10, eotaxin, MIG and TNFR-II are higher in older subjects in contrast to basal IL-6 |
| [6] | 55-89 | Caregivers=119 C=106 | H | L | Multi-level model to analyze predictive factor for slope.  Adjusted for cormorbidities | Basal IL-6 increases with age and chronical stress but stroke, type 2 diabetes or cancer do not explain slope |
| [7] | m=36-59  y=20-35  o>60 | o=50 m=25 y=15 | S and H | T | T test and Cochran cox test | IL-6 in the sera and spontaneous secretion of PBMC of Il-6 are associated with bad health status and age |
| [8] | >75 | LOAD=60 VD=80 | Day hospital patient ; Barthel index (BI) | T | MVA adjusted for age, BI, gender, CV risk factors and diseases, atrial fibrillation. | IL-6 is positively correlated with age and negatively correlated with functional status |
| [9] | >20 | n=1327 | H | T | linear regression models adjusted for subclinical cardiovascular diseases, CV risk factors and chronically diseases | Increase of IL-6 and CRP with age and cardiovascular diseases |
| [10] | 70-79 | n=2225 | C | L | Cox proportional hazard adjusted for age, gender, comorbidities, CV risk factors and diseases | TNFα, IL-6 and CRP are independent predictors of cardiovascular diseases |
| [11] | > 65 | n=1016 | H, MMSE and DSM IV | L | Cox hazard regression models adjusted for CV risk factors, NSAI and statin intake | No inflammatory markers predict AD but combination of high IL-6 and CRP predicts vascular dementia |
| [12] | 55-94 | LOAD=60 VD= 60 C=33 | defined by neurologist for dementia, MMSE | T | MVA adjusted for age only | No difference for TNFα and Il-1β between groups |
| [13] | 70-80 | LOAD= 60 VD=80 Stroke=40 C=42 | defined by neurologist and MMSE | T | MVA adjusted for age, gender, CV diseases and risk factors. | No difference for IL-10; Il-1β is higher in VD, LOAD, CDND compared to control; TNFα is higher is VD and LOAD compare to control; IL-6 is higher in VA compare to LOAD |
| [14] | 74-80 | Alzheimer=145; C=51 | N and C | T | Mann-Whitney rank sum test; no adjustement for CV diseases | IL-6 is increased in Alzheimer patients |
| [15] | 65-79 | N=3298 | C | T | MVA adjusted for age, gender, arterial hypertension, CV diseases and risk factors | IL-6 levels is negatively correlated with MMSE |
| [16] | 70-79 | N=724 | C and disabled women , Fried | T | MVA adjusted for age, smoking, BMI, type 2diabetes, congestive heart failure | Highest tertile of IL-6 correlates with frailty; CMV serology is not significantly associated with IL-6 levels |
| [17] | >65 | 4735 | C; Fried | T | MVA adjusted for age, gender, race and CV diseases | High IL-6 and CRP levels are significantly associated with frailty |
| [18] | >55 | n=986 | H | L | MVA adjusted educational level, smoking status, number of chronic disease, alcohol use, physical activity, NSAI, BMI, cognitive impairment and depressive symptoms | Increased IL-6 is associated with decrease of muscle strength. No association for CRP |
| [19] | >74 | frail=11 C =19 | H, Fried criteria | T | Mann-Whitney rank sum test and Pearson correlation, no adjustment | Higher IL-6 levels in frail patients |
| [20] | 70-79 | n= 2979 | H | L | Cox hazards regression models adjusted for age, gender, race, education, comorbidities, CV risk factors and diseases, NSAI and corticoids intake | CRP, IL-6 and TNFα predict an increased incidence of mobility limitation during 30 months follow-up especially in subjects with high levels of all 3 markers |
| [21] | >65 | n=620 W | H, ADL, walking speed, knee strength | L | MVA, Cox proportional hazard models adjusted for BMI, race, smoking, heart failure, coronary diseases, Peripheral artery diseases, COPD, type 2 diabetes, osteoarthritis | Increase of IL-6 levels is associated with decreased mobility, ADL and severe walking disability |
| [22] | >65 | n=77 | N, grip strength, Tinetti, Mob-T, Elderly mobility scale | T | ANCOVA | High IL-6 and Hsp70 are correlated with worse muscle endurance |
| [23] | 70-79 | C=2879 Depressed =145 | H; depression scale | T | MVA adjusted for gender, age, total fat mass, diseases, NSAI, antidepressant intake, smoking and alcohol use | High CRP, IL-6 and TNFα levels are associated with higher risk of depressed mood. |
| [24] | 70-80 | n=40 | H ; exclusion of CRP>0,3mg/dl | T | Spearman correlation unadjusted | High IL-6 levels are positively correlated with fatigue resistance and grip work |
| [25] | 50-78 | caregivers=107 C=143 | H ; self rated health | T | MVA adjusted for age, BMI, gender, number of health conditions | Poorer self rated health was significantly associated with high IL-6 and CRP levels and age |
| [26] | 70-90 | n=1037 | C ; GDS | L | MVA adjusted for age, gender, education, number of diseases, CV diseases, endocrine disorder, NSAI, antidepressant, statin intake | IL-6 levels are associated with depressive symptoms at baseline only, Il-8 levels at baseline and follow up |
| [27] | >65 | n=120 | N ; Katz and BI | T | linear regression models adjusted for subclinical cardiovascular diseases, CV risk factors and chronically diseases, AINS | IL-6 and soluble TNFreceptor are associated with BI and Katz scores in contrast to IL-1β |
| [28] | 20-72 | n=220 W | C ; grip strength and walking performance  Exclusion of ischemic heart diseases | T | MVA adjusted for age, menopausal status, body height, grip strength, cholesterol, creatinin clearance, smoking, 25(OH)vitD | IL-6 levels are associated with reduced walking performance |
| [29] | ≥ 65 | n= 301 | community acquired pneumonia | L | Cox proportional ratio and MVA adjusted for Charlson index, MMSE, ADL | TNFα levels are associated with functional decline but not with hospital readmission or death. CRP is not significantly correlated with these outcomes. |
| [30] | >65 | n= 970 | C, postmenopausal women, Fried criteria | L | Logistic regression adjusted for hypertension, smoking, BMI, hormonal use, education, arthritis | Il-6 and CRP levels are not associated with frailty |
| [31] | >85 | n=1622 | H and N; Fried criteria | T | Logistic regression adjusted for number of comorbidities, corticoid and NSAI use | High CRP and spontaneous secretion of IL-6 by PBMC are correlated with frailty |
| [32] | 70-79 years and >65 y | N=704 | H and disabled in only one functional domain, fried criteria | T | Logistic regression adjusted for CV diseases, arthritis, cancer,chronic diseases | IL-6 levels, alone, are not associated with frailty but combination of anemia, IL-6, IGF1, DHEA, micronutrient deficits are associated with frailty. |
| [33] | >65 | N=1509 | Frail ; non frail assessed by BMI, low peak flow, MMSE, hearing and vision problems, urinary incontinence, depression and physical activity | L&T | Logistic regression analyses adjusted for CV diseases or risk factors, cancer, chronic disease, NSAI and estrogen use | Transversally, CRP and IL-6 levels are not associated with frailty in contrast to 25(OH) vitD  Longitudinally, CRP and 25(OH) vit D are associated with incidence of frailty |

Age in year; o= old people; m= middle aged; y= young people; L= longitudinal study; T = Transversal study; H= healthy or community dwelling; S= senieur protocol; N= nursing home patients; PEF= peak expiratory flow; MVA= multivariate analyses; LOAD = late onset Alzheimer disease; VD= vascular dementia; C= controls; W= women; M= men, NSAI= non steroid anti-inflammatory drugs

**B) Inflammatory markers as predictive factor for mortality in old age**

| Reference | Age | Group size | Clinical characteristics | Design | Statistics | Outcomes |
| --- | --- | --- | --- | --- | --- | --- |
| [34] | >100 | n=126 | N or H | L | Cox regression model adjusted for dementia, CV diseases. | TNFα and CRP were associated with mortality but not IL-6 |
| [35] | > 65 | M= 153 W= 101 | H, Fried criteria | L | cox regression model adjusted for baseline age, gender, social class, smoking, weight, number of system medicated | IL-1β is associated with mortality but not when adjusted for confounding variable, no association with mortality and fraily for Il-6 and CRP |
| [36] | >80 | n=362 | H and N ; ADL, comorbidities | L | Cox regression model for gender, CV risk factors and diseases, chronic disease | No relation between circulating cytokines and prevalence of chronic diseases, but with BMI, smoking and physical inactivity. High IL-6 but not TNFα is associated with mortality |
| [37] | 70-79 | n=870 | N and H | L | MVA adjusted for age, gender, education, CV disease and risk factors | CRP and IL-6 levels predict 3- and 7-years mortality |
| [38] | >80 | n=362 | N | L | cox proportional hazard adjusted for age, gender, ADL, cognitive performance test, number of diseases, hearing impairment, albumin, cholesterol, BMI | low IL-6, TNFα, CRP are associated with better survival |
| [39] | 72-92 | n=525 | C | L | Kaplan Meier and proportional hazard adjusted for age, gender, arthritis, CV disease and risk factors | Elevated Serum IL-6,decreased IGF-1 and elevated PBMC production of TNFα are all associated with mortality at 4 years but not IL-1β or PBMC production of IL-6 |
| [40] | >65 | n=1723 | C ; ADL, IADL, Katz | L | Hazard proportional model, MVA adjusted for age, gender, race, chronic disease, functional status at baseline | IL-6 and D-dimer are both associated with mortality and functional dependence |
| [41] | >70 | n=137 W | Center for memory disorder | T | Spearman correlation adjusted for age and systolic blood pressure | IL-6 is associated with development of leukoaraiosis. |
| [42] | >65 | N=3673 | C | L | Hazard ratio adjusted for age, CV risk factors and diseases. | IL-6 and CRP levels are associated with mortality |
| [43] | >58 | N=1834 | H; | L | COX proportional hazard regression adjusted for comorbidites | IL-6 is associated with all cause mortality, CVD mortality |
| [44] | 70-79 | N=2234 | H | L | Cox proportional hazard regression adjusted age, gender, CV risk factors and disease, cancer, chronic disease, AINS, statins, corticoids use | IL-6 is associated with disability, mortality and impaired mobility ; IL-6 and oxidative markers are associated with mortality |
| [45] | ≥65 | N=1677 | C ; assessed for cognitive trouble, gait speed, grip strength | L | Wilcoxon rank sum test, chi², multivariate regression adjusted for age, gender, ethnicity, smoking status, dyslipidemia, BMI, hypertension and CV diseases | Increasing levels of IL-6 are significantly associated with mobility and cognitive trouble, incidence of CV diseases and mortality |

**References**

1. Miles EA, Rees D, Banerjee T, Cazzola R, Lewis S, et al. (2008) Age-related increases in circulating inflammatory markers in men are independent of BMI, blood pressure and blood lipid concentrations. Atherosclerosis 196: 298-305.

2. Forsey RJ, Thompson JM, Ernerudh J, Hurst TL, Strindhall J, et al. (2003) Plasma cytokine profiles in elderly humans. Mech Ageing Dev 124: 487-493.

3. Della Bella S, Bierti L, Presicce P, Arienti R, Valenti M, et al. (2007) Peripheral blood dendritic cells and monocytes are differently regulated in the elderly. Clin Immunol 122: 220-228.

4. Beharka AA, Meydani M, Wu D, Leka LS, Meydani A, et al. (2001) Interleukin-6 production does not increase with age. J Gerontol A Biol Sci Med Sci 56: B81-B88.

5. Shurin GV, Yurkovetsky ZR, Chatta GS, Tourkova IL, Shurin MR, et al. (2007) Dynamic alteration of soluble serum biomarkers in healthy aging. Cytokine 39: 123-129.

6. Kiecolt-Glaser JK, Preacher KJ, MacCallum RC, Atkinson C, Malarkey WB, et al. (2003) Chronic stress and age-related increases in the proinflammatory cytokine IL-6. Proc Natl Acad Sci U S A 100: 9090-9095.

7. Mysliwska J, Bryl E, Foerster J, Mysliwski A (1998) Increase of interleukin 6 and decrease of interleukin 2 production during the ageing process are influenced by the health status. Mech Ageing Dev 100: 313-328.

8. Zuliani G, Guerra G, Ranzini M, Rossi L, Munari MR, et al. (2007) High interleukin-6 plasma levels are associated with functional impairment in older patients with vascular dementia. Int J Geriatr Psychiatry 22: 305-311.

9. Ferrucci L, Corsi A, Lauretani F, Bandinelli S, Bartali B, et al. (2005) The origins of age-related proinflammatory state. Blood 105: 2294-2299.

10. Cesari M, Penninx BW, Newman AB, Kritchevsky SB, Nicklas BJ, et al. (2003) Inflammatory markers and onset of cardiovascular events: results from the Health ABC study. Circulation 108: 2317-2322.

11. Ravaglia G, Forti P, Maioli F, Chiappelli M, Montesi F, et al. (2007) Blood inflammatory markers and risk of dementia: The Conselice Study of Brain Aging. Neurobiol Aging 28: 1810-1820.

12. Yasutake C, Kuroda K, Yanagawa T, Okamura T, Yoneda H (2006) Serum BDNF, TNF-alpha and IL-1beta levels in dementia patients: comparison between Alzheimer's disease and vascular dementia. Eur Arch Psychiatry Clin Neurosci 256: 402-406.

13. Zuliani G, Ranzini M, Guerra G, Rossi L, Munari MR, et al. (2007) Plasma cytokines profile in older subjects with late onset Alzheimer's disease or vascular dementia. J Psychiatr Res 41: 686-693.

14. Licastro F, Pedrini S, Caputo L, Annoni G, Davis LJ, et al. (2000) Increased plasma levels of interleukin-1, interleukin-6 and alpha-1-antichymotrypsin in patients with Alzheimer's disease: peripheral inflammation or signals from the brain? J Neuroimmunol 103: 97-102.

15. Wright CB, Sacco RL, Rundek TR, Delman JB, Rabbani LE, et al. (2006) Interleukin-6 is associated with cognitive function: the Northern Manhattan Study. J Stroke Cerebrovasc Dis 15: 34-38.

16. Schmaltz HN, Fried LP, Xue QL, Walston J, Leng SX, et al. (2005) Chronic cytomegalovirus infection and inflammation are associated with prevalent frailty in community-dwelling older women. J Am Geriatr Soc 53: 747-754.

17. Walston J, McBurnie MA, Newman A, Tracy RP, Kop WJ, et al. (2002) Frailty and activation of the inflammation and coagulation systems with and without clinical comorbidities: results from the Cardiovascular Health Study. Arch Intern Med 162: 2333-2341.

18. Schaap LA, Pluijm SM, Deeg DJ, Visser M (2006) Inflammatory markers and loss of muscle mass (sarcopenia) and strength. Am J Med 119: 526-17.

19. Leng S, Chaves P, Koenig K, Walston J (2002) Serum interleukin-6 and hemoglobin as physiological correlates in the geriatric syndrome of frailty: a pilot study. J Am Geriatr Soc 50: 1268-1271.

20. Penninx BW, Kritchevsky SB, Newman AB, Nicklas BJ, Simonsick EM, et al. (2004) Inflammatory markers and incident mobility limitation in the elderly. J Am Geriatr Soc 52: 1105-1113.

21. Ferrucci L, Penninx BW, Volpato S, Harris TB, Bandeen-Roche K, et al. (2002) Change in muscle strength explains accelerated decline of physical function in older women with high interleukin-6 serum levels. J Am Geriatr Soc 50: 1947-1954.

22. Bautmans I, Njemini R, Predom H, Lemper JC, Mets T (2008) Muscle endurance in elderly nursing home residents is related to fatigue perception, mobility, and circulating tumor necrosis factor-alpha, interleukin-6, and heat shock protein 70. J Am Geriatr Soc 56: 389-396.

23. Penninx BW, Kritchevsky SB, Yaffe K, Newman AB, Simonsick EM, et al. (2003) Inflammatory markers and depressed mood in older persons: results from the Health, Aging and Body Composition study. Biol Psychiatry 54: 566-572.

24. Bautmans I, Gorus E, Njemini R, Mets T (2007) Handgrip performance in relation to self-perceived fatigue, physical functioning and circulating IL-6 in elderly persons without inflammation. BMC Geriatr 7: 5.

25. Christian LM, Glaser R, Porter K, Malarkey WB, Beversdorf D, et al. (2011) Poorer self-rated health is associated with elevated inflammatory markers among older adults. Psychoneuroendocrinology 36: 1495-1504.

26. Baune BT, Smith E, Reppermund S, Air T, Samaras K, et al. (2012) Inflammatory biomarkers predict depressive, but not anxiety symptoms during aging: The prospective Sydney Memory and Aging Study. Psychoneuroendocrinology .

27. Gonzalo-Calvo D, Luxan-Delgado B, Rodriguez-Gonzalez S, Garcia-Macia M, Suarez FM, et al. (2012) Interleukin 6, soluble tumor necrosis factor receptor I and red blood cell distribution width as biological markers of functional dependence in an elderly population: a translational approach. Cytokine 58: 193-198.

28. Blain H, Jaussent A, Beziat S, Dupuy AM, Bernard PL, et al. (2012) Low serum IL-6 is associated with high 6-minute walking performance in asymptomatic women aged 20 to 70years. Exp Gerontol 47: 143-148.

29. El Solh A, Pineda L, Bouquin P, Mankowski C (2006) Determinants of short and long term functional recovery after hospitalization for community-acquired pneumonia in the elderly: role of inflammatory markers. BMC Geriatr 6: 12.

30. Reiner AP, Aragaki AK, Gray SL, Wactawski-Wende J, Cauley JA, et al. (2009) Inflammation and thrombosis biomarkers and incident frailty in postmenopausal women. Am J Med 122: 947-954.

31. Collerton J, Martin-Ruiz C, Davies K, Hilkens CM, Isaacs J, et al.(2012) Frailty and the role of inflammation, immunosenescence and cellular ageing in the very old: cross-sectional findings from the Newcastle 85+ Study. Mech Ageing Dev 133: 456-466.

32. Fried LP, Ferrucci L, Darer J, Williamson JD, Anderson G (2004) Untangling the concepts of disability, frailty, and comorbidity: implications for improved targeting and care. J Gerontol A Biol Sci Med Sci 59: 255-263.

33. Puts MT, Visser M, Twisk JW, Deeg DJ, Lips P (2005) Endocrine and inflammatory markers as predictors of frailty. Clin Endocrinol (Oxf) 63: 403-411.

34. Bruunsgaard H, Andersen-Ranberg K, Hjelmborg JB, Pedersen BK, Jeune B (2003) Elevated levels of tumor necrosis factor alpha and mortality in centenarians. Am J Med 115: 278-283.

35. Baylis D, Bartlett DB, Syddall HE, Ntani G, Gale CR, et al. (2012) Immune-endocrine biomarkers as predictors of frailty and mortality: a 10-year longitudinal study in community-dwelling older people. Age (Dordr ) .

36. Bruunsgaard H, Ladelund S, Pedersen AN, Schroll M, Jorgensen T, et al. (2003) Predicting death from tumour necrosis factor-alpha and interleukin-6 in 80-year-old people. Clin Exp Immunol 132: 24-31.

37. Reuben DB, Cheh AI, Harris TB, Ferrucci L, Rowe JW, et al. (2002) Peripheral blood markers of inflammation predict mortality and functional decline in high-functioning community-dwelling older persons. J Am Geriatr Soc 50: 638-644.

38. Giovannini S, Onder G, Liperoti R, Russo A, Carter C, et al. (2011) Interleukin-6, C-reactive protein, and tumor necrosis factor-alpha as predictors of mortality in frail, community-living elderly individuals. J Am Geriatr Soc 59: 1679-1685.

39. Roubenoff R, Parise H, Payette HA, Abad LW, D'Agostino R, et al. (2003) Cytokines, insulin-like growth factor 1, sarcopenia, and mortality in very old community-dwelling men and women: the Framingham Heart Study. Am J Med 115: 429-435.

40. Cohen HJ, Harris T, Pieper CF (2003) Coagulation and activation of inflammatory pathways in the development of functional decline and mortality in the elderly. Am J Med 114: 180-187.

41. Nagai K, Kozaki K, Sonohara K, Akishita M, Toba K (2011) Relationship between interleukin-6 and cerebral deep white matter and periventricular hyperintensity in elderly women. Geriatr Gerontol Int 11: 328-332.

42. Harris TB, Ferrucci L, Tracy RP, Corti MC, Wacholder S, et al. (1999) Associations of elevated interleukin-6 and C-reactive protein levels with mortality in the elderly. Am J Med 106: 506-512.

43. Lee JK, Bettencourt R, Brenner D, Le TA, Barrett-Connor E, et al. (2012) Association between serum interleukin-6 concentrations and mortality in older adults: the Rancho Bernardo study. PLoS One 7: e34218.

44. Cesari M, Kritchevsky SB, Nicklas B, Kanaya AM, Patrignani P, et al. (2012) Oxidative damage, platelet activation, and inflammation to predict mobility disability and mortality in older persons: results from the health aging and body composition study. J Gerontol A Biol Sci Med Sci 67: 671-676.

45. Jenny NS, French B, Arnold AM, Strotmeyer ES, Cushman M, et al. (2012) Long-term assessment of inflammation and healthy aging in late life: the Cardiovascular Health Study All Stars. J Gerontol A Biol Sci Med Sci 67: 970-976.
